# Reporting baseline variables – descriptive summaries rather than inferential comparisons: editorial series on improving reporting and methodological rigor in scientific articles

**DOI:** 10.2340/17453674.2026.46365

**Published:** 2026-07-02

**Authors:** Robin CHRISTENSEN, Aleksi REITO, Tobias HAUGEGAARD, Søren OVERGAARD

**Affiliations:** 1 Section for Biostatistics and Evidence-Based Research, the Parker Institute, Bispebjerg and Frederiksberg Hospital, Copenhagen, Denmark; 2 Research Unit of Rheumatology, Department of Clinical Research, University of Southern Denmark, Odense University Hospital, Denmark; 3 Center for Musculoskeletal Diseases, Tampere University Hospital, Tampere; 4 Coxa Hospital for Joint Replacement, Tampere, Finland; 5 Department of Orthopaedic Surgery and Traumatology, Copenhagen University Hospital, Bispebjerg, Denmark; 6 Department of Clinical Medicine, Faculty of Health and Medical Sciences, University of Copenhagen, Copenhagen, Denmark

## Abstract

Baseline characteristics describe the pre-exposure or pre-intervention properties of a study sample and are intended to inform interpretation and the assessment of comparability, rather than to support inferential comparisons beyond what was observed. Baseline variables should be pre-specified and justified in the study protocol, guided by clinical relevance, established reporting standards and frameworks, and presented in a dedicated table following the study flow diagram. Continuous variables should be summarized as means with standard deviations or, when skewed, medians with interquartile ranges; categorical variables as counts with proportions, reported separately by comparison group with absolute numbers and indication of missingness. In randomized trials, significance testing of baseline differences is not recommended, as any observed differences arise by chance. In cohort studies, baseline comparisons should be interpreted descriptively as evaluations of group comparability and the potential for confounding; standardized differences are preferred for quantifying imbalance. By complementing existing reporting standards and addressing common deficiencies, this guidance supports consistent, reproducible reporting of baseline variables and clearer communication of study populations.

Descriptive statistics summarize the observed data using numerical and graphical methods to characterize central tendency, variability, and distribution, without drawing inferences beyond the sample. In contrast, statistical inference uses sample data to draw conclusions about a broader population and quantifies the uncertainty associated with those conclusions. Accordingly, baseline characteristics represent descriptive properties of the study sample intended to inform interpretation and assessment of comparability, rather than to support inferential comparisons beyond what was simply observed in the acquired sample.

This principle is most clearly illustrated in the reporting of baseline characteristics, typically presented as in Table 1. Baseline characteristics describe participant attributes measured before exposure or intervention. In observational studies and randomized trials, they show what the study population looked like at the outset and provide a transparent account of demographic, clinical, and other relevant pre-exposure variables without implying inference or group comparison [1]. This includes contextual factors, defined as variables that are not outcomes but must be recognized and measured to understand study results in the appropriate context, including factors that influence prognosis, modify treatment effects, or affect measurement [2].

**Table 1A:** Template for a baseline characteristics table in a randomized trial. Data should be reported as mean (SD), unless otherwise specified

Item	Experimental Intervention (n = ??)	Control Comparator (n = ??)	Total ^a^ (N = ???)
Characteristic			
Age, years			
Female sex, n (%)			
Body-mass index			
Educational level above high school, n (%)			
Outcome measures (baseline):			
Oxford Hip Score			
Pain			
Symptoms			
Function in activities of daily living			
Hip-related quality of life			
Function in sports and recreation			
UCLA activity score			

a The total column enhances interpretability by explicitly presenting the average characteristics of the study population, rather than requiring readers to infer them from group-specific summaries.

Baseline characteristics should be presented in a dedicated table describing the pre-exposure or pre-intervention properties of the study population [3]. In standard biomedical reporting, and in *Acta Orthopaedica* in particular, this table typically follows the study flow diagram because the flow diagram defines the analysis population (i.e., who was included, excluded, and analyzed), whereas the baseline table characterizes the population to which the subsequent findings apply. The flow diagram defines the analysis population, whereas the baseline table characterizes it. Presenting these elements in sequence allows readers to verify the sample’s composition and comparability. This order follows the reporting logic from sample identification to sample description [3]—and facilitates transparent interpretation of the analysis population.

## Baseline variable selection

Baseline variables should be prespecified and justified in the study protocol, guided by clinical relevance and established reporting standards. They should capture factors important for interpretation, applicability, and decision-making, and reflect input from relevant interest-holders to ensure relevance and transparency [4]. In this way, baseline variable selection is not an ad hoc exercise, but should be perceived as a protocol-driven step aligned with the study’s estimand, design, and target population, ensuring both methodological rigor and relevance. The Outcome Measures in Rheumatology (OMERACT) Contextual Factors Working Group provides a structured framework, identifying candidate factors across 3 domains: personal (e.g., age, sex, socioeconomic status, lifestyle), disease-related (e.g., disease severity, duration, comorbidities, mental health), and environmental (e.g., healthcare access, social support, patient–provider interaction) [5]. These considerations guide interpretation but do not define a fixed set of variables. *Acta Orthopaedica* recommends that baseline summaries include relevant demographic and clinical characteristics reported using appropriate descriptive statistics to characterize the study population [1,3].

## Reporting guideline

As already indicated, reporting baseline characteristics is a core requirement for transparent study reporting across designs [3]. Guidance for both observational studies [6] and randomized trials [7] specifies that participant characteristics should be presented in a dedicated table within the Results section. Typically placed immediately after the study flow diagram, the baseline table (Table 1) summarizes key variables by comparison group. Its purpose is to characterize the analysis population, assess group comparability, and support evaluation of internal validity and generalizability.

**Table 1B:** Template for a baseline characteristics table in a cohort study. Data should be reported as mean (SD), unless otherwise specified

Item	Exposed group (n = ??)	Unexposed/Control group (n = ??)	Standardized difference ^a^ (N = ???)
Characteristic			
Age, years			
Female sex, n (%)			
Body-mass index			
Educational level above high school, n (%)			
Outcome measures (baseline):			
Oxford Hip Score			
Pain			
Symptoms			
Function in activities of daily living			
Hip-related quality of life			
Function in sports and recreation			
UCLA activity score			

a Standardized differences are calculated as the difference in means or proportions between groups divided by the pooled standard deviation, providing a unitless measure of imbalance independent of sample size.

Continuous variables should be reported as means with standard deviations when approximately normally distributed and as medians with interquartile ranges when skewed. Categorical variables should be presented as counts with proportions (%). Data should be reported separately by comparison group, with absolute numbers provided alongside percentages and, where relevant, the number of observations for each variable to indicate missingness. Baseline summaries should not include confidence intervals or standard errors and should not be subjected to hypothesis testing, as they reflect observed sample properties and are not intended for statistical inference. This principle is particularly emphasized in randomized trials, where any observed differences between groups are expected to arise by chance [7]. Accordingly, *Acta Orthopaedica* concurs with what is explicitly stated in the CONSORT guidance: “significance testing of baseline differences is not recommended and should not be reported,” as any observed differences between randomized groups arise by chance rather than bias.

In cohort studies, group comparisons at baseline should not rely on hypothesis testing. Baseline differences reflect possible confounding rather than chance, and meaningful interpretation requires estimation rather than P values, which are driven by sample size and do not quantify imbalance [6,8]. In cohort studies, baseline comparisons should be interpreted as descriptive evaluations of group comparability, informing the potential for confounding rather than serving as formal statistical tests. Differences are better quantified using standardized differences, which express contrasts in means for continuous variables and contrasts in prevalences or proportions for binary variables relative to the pooled standard deviation and are therefore not inherently influenced by sample size [9].

Consequently, *Acta Orthopaedica* encourages authors reporting cohort studies to use standardized differences, which express between-group differences in standard deviation units. These measures are applicable to both continuous and binary variables and quantify the magnitude of imbalance relative to the natural variability of the observed data. Because similarity in means or proportions alone does not guarantee comparable underlying distributions, assessment should extend beyond measures of central tendency to include variability, distributional characteristics, and, where appropriate, graphical diagnostics. As a practical heuristic, standardized differences below approximately 0.10 are often considered negligible, although interpretation should account for the prognostic importance of each variable.

## Closure

In line with the principles of this editorial series, this guidance complements existing reporting standards by addressing common deficiencies and providing practical, design-specific recommendations to improve clarity, transparency, and interpretability. We hope it will assist authors in achieving consistent and reproducible reporting of baseline variables, enabling clearer communication of study populations and more reliable interpretation of research findings.
